# Performance of Single-Layer Lining Using Shotcrete and Reinforcement Ribs Employed for Supporting Large-Span Tunnel

**DOI:** 10.3390/ma16247590

**Published:** 2023-12-11

**Authors:** Zijian Li, Pinxin Diao, Weihua Lu, Yongxing Zhang

**Affiliations:** School of Civil Engineering, Nanjing Forestry University, Nanjing 210037, China

**Keywords:** performance, single-layer lining, shotcrete, reinforcement ribs, large-span tunnel

## Abstract

Single-layer tunnel lining using shotcrete has the significant advantages of reducing the tunnel excavation volume and saving construction materials, which has been gradually applied in tunnel construction. The high-performance concretes are generally adopted in single-layer tunnel lining for enhancing the bearing capacity of the tunnel lining, whereas the single-layer tunnel lining may still induce damages due to the adverse conditions such as shallow buried depth of the tunnel, and further study related to its application condition is thus required. This paper presents a study of the single-layer tunnel lining with shotcrete employed for supporting the large-span tunnel, in which the reinforcement ribs are also adopted in the single-layer lining for improving the lining stiffness and strength. The study is implemented using numerical simulation, focusing on the safety and performance variation of the single-layer tunnel lining influenced from the varied lining thicknesses and shallow buried depths of the tunnel. The results shows that the single-layer tunnel lining has the obvious advantage of toughness in significantly absorbing large deformation of surrounding rocks and improving the ability to resist lining cracking. The results also demonstrate that the single-layer tunnel lining with shotcrete and reinforcement ribs can safely support the large-span tunnel, in which the stability and safety of the large-span tunnel are confirmed from both the tunnel deformation and lining stresses. Moreover, the factors related to both the lining thickness and shallow buried depth of the tunnel have great influence on the single-layer tunnel lining with shotcrete and reinforcement ribs, in which the insufficient lining thickness and excessive shallow buried depth of the tunnel can induce the damages of the single-layer tunnel lining due to shotcrete stresses exceeding its strength. This study provides some references of employing the single-layer tunnel lining with shotcrete and reinforcement ribs for supporting large-span tunnel.

## 1. Introduction

Tunnel has been widely developed in transportation infrastructure, in which the lining structure is significantly important for supporting tunnel excavation [1,2], and the composite lining structure of the primary lining-waterproof layer-secondary lining are conventionally adopted in tunnel construction [3]. However, there are obvious shortages of the aforementioned composite lining structure with various tunnel lining defects [4], in which the poor fit between the primary lining and secondary lining easily worsens the stress conditions of the composite lining structure and weakens the supporting effect of the composite lining structure for the stability of surrounding rocks [5]. Moreover, the flexible waterproof layer in the composite lining structure easily forms the deficiency of water storage space with water pressure and induces the lining cracking and leakage [6,7]. Especially, it is extremely uneconomical to still apply the aforementioned composite lining structure for supporting the surrounding rocks with good condition, since the secondary lining is usually adopted as the safety reserve in the composite lining structure. Therefore, there is an urgent requirement to develop more economical and applicable lining structure in tunnel construction.

In view of previous studies, shotcrete adopted in lining structure can increase the overall performance of the tunnel lining for supporting the surrounding rocks [8,9,10], which can efficiently and timely bond well to most substrates [11,12,13]. Moreover, the high-performance concrete has been gradually applied as shotcrete in building the tunnel lining for enhancing the tunnel performance in recent years [14,15,16]. The fiber-reinforced concrete (FRC) is adopted in tunnel lining for increasing the stability and bearing capacity of surrounding rocks [17,18,19], the functional cementitious composite (FCC) and fiber-reinforced plastics (FRP) are utilized to increase the deformation ability and damage tolerance of lining structure [20,21,22], and steel plate is used for resisting the explosion impact on tunnel lining [23]. This application of the high-performance materials provides the opportunity of developing new-type lining structure without the shortages in the aforementioned composite lining structure, in which the single-layer tunnel lining using shotcrete is developed for supporting the tunnel construction [15].

In recent years, single-layer tunnel lining using shotcrete has been gradually applied in tunnel construction, which has the significant advantages of reducing the tunnel excavation volume. Moreover, the single-layer tunnel lining using shotcrete can significantly reduce material consumption with good economic efficiency, and it is easily to take remedial measures with the reserved reinforcement space when the tunnel diseases occur. Especially, the high-performance concretes are generally adopted in single-layer tunnel lining for enhancing the bearing capacity of the tunnel lining [24]. However, the single-layer tunnel lining using shotcrete may still induce damages with the adverse conditions such as shallow buried depth of the tunnel, and further study related to its application condition is urgently required. This paper presents a study of single-layer tunnel lining with shotcrete employed for supporting the large-span tunnel, in which the reinforcement ribs are also adopted in the single-layer lining for improving the lining stiffness and strength. The study is implemented by numerical analysis, focusing on the safety and performance variation of the single-layer tunnel lining influenced from the varied lining thicknesses and shallow buried depths of the tunnel. Moreover, the axial forces and bending moment of the typical parts in the single-layer tunnel lining and the traditional composite lining structure are compared for verifying the practicability of employing the single-layer tunnel lining for supporting the large-span tunnel in tunnel construction engineering.

## 2. Engineering Background

### 2.1. Engineering Profile

The large-span tunnel has 331 m total longitudinal length with 12.2 m excavation span in the tunnel section. According to the field investigated geological data around the tunnel site, the tunnel is located at the karst mountainous area with the exposed bedrock, in which the terrain slope of the mountain greatly varies from 25° to 55°, and the rock mass is relatively broken without developed fissures and gullies on the ground. The tunnel mainly passes through the limestone formations with hard rock quality, in which the surrounding rocks has good integrity and strong self-stabilization. Moreover, the minimum and maximum buried depths of the tunnel are 39 m and 55 m respectively, in which the tunnel entrance crosses the lower part of the mountain slope, and the tunnel exit is located above the slope of the mountain. 

### 2.2. Single-Layer Tunnel Lining Using Shotcrete and Reinforcement Ribs

**Figure 1:**
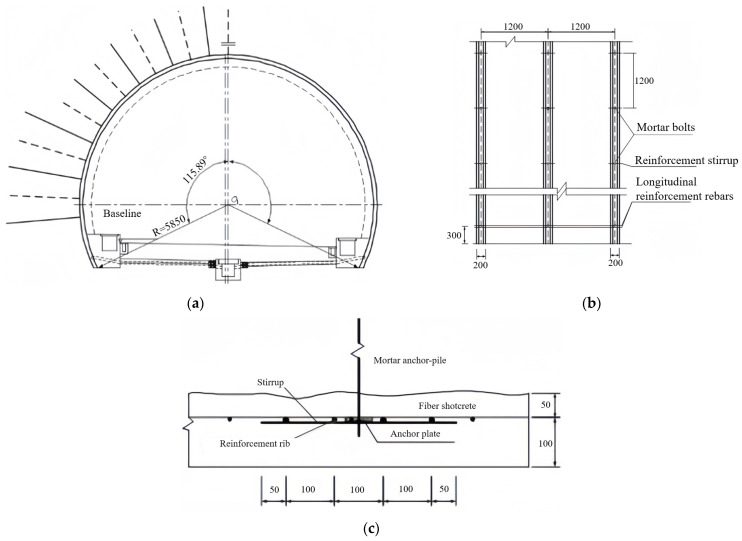
Single-layer lining using shotcrete and reinforcement tibs: (**a**) Section map; (**b**) Reinforcement ribs; (**c**) Connection of reinforcemnt ribs and bolts (unit: mm).

Figure 1 shows the cross-sectional view of the single-layer tunnel lining using shotcrete and reinforcement ribs, in which the single-layer tunnel lining is constituted of the shotcrete using steel fiber reinforced concrete with 0.2 m thickness, reinforcement ribs and system bolts. The shotcrete has 0.15 m thickness as well as 16.7 MPa compressive strength and 1.78 MPa tensile strength. The reinforcement rib is formed by three 0.06 m-diameter rebars with 0.25 m spacing, which is arranged as 1.2 m spacing in longitudinal direction. Moreover, the mortar bolts with 2.0 m length and 3.0 m length are respectively arranged within tunnel arch and side walls, which are fixed with 1.2 m × 1.2 m spacing in longitudinal and circumferential directions.

## 3. Proposal and Validation of the Numerical Investigation Model

### 3.1. Numerical Models and Material Properties

**Figure 2:**
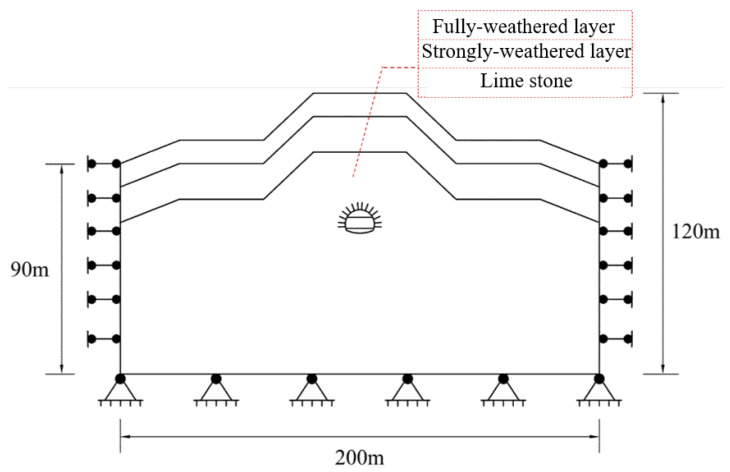
Numerical model and boundary conditions.

The behavior of the single-layer tunnel lining using shotcrete and reinforcement ribs for supporting the large-span tunnel is numerically studied by employing the commercial software Midas with finite element method. The study is expected to confirm the safety of the single-layer tunnel lining for supporting the large-span tunnel, which also can clarify the performance variation of the single-layer tunnel lining influenced from the varied lining thicknesses and shallow buried depths of the tunnel. Figure 2 shows the numerical model and boundary condition. In the numerical model, the horizontal calculation range is 200 m, the vertical calculation ranges from top surface to lower boundary is 120 m, including 90 m for that of both lateral sides in the vertical direction. The bottom boundary is fixed in both longitudinal and vertical directions, and both lateral boundaries are constrained in longitudinal direction, whereas the upper boundary is free in both longitudinal and vertical directions. Moreover, the calculation of ground stress balance is executed and the calculated displacement is returned to zero for removing the adverse influence on sliding surface.

In the numerical model, the excavation span of the tunnel section is 12.2 m, and the buried depth of the tunnel is 50 m. The stratum in the model is divided into three layers starting from the ground surface and similar with that observed in field, in which the full weathering layer has 10 m thickness, strong weathering layer has 15 m thickness, and the below one is limestone layers. The construction method with upper and lower benches is employed for tunnel excavation, and the single-layer lining is added after tunnel excavation.

The solid and beam elements with small mesh sizes are respectively adopted to simulate the surrounding rocks and single-layer tunnel lining, which is expected to ensure the accuracy of the numerical result. Moreover, the surrounding rocks is considered as ideal elastic-plastic material with Mohr–Coulomb yield criterion [25,26], and the material parameters of the surrounding rocks and single-layer tunnel lining are respectively shown in Table 1 and Table 2.

The surrounding rocks are considered as ideal elastic-plastic material met with Mohr-Coulomb yield criterion, which can also reflect the behavior of surrounding rocks during tunneling. Moreover, the effect of reinforcement ribs in single-layer lining is consider by using the principle of equivalent stiffness, in which the elastic modulus of reinforcement ribs is converted to that of shotcrete by the following calculation formula [27]:

(1) $$E^{\prime }=E_{0}+\frac{S_{g}}{S_{c}}E_{g}$$

where *E*′ is equivalent Young’s modulus; *E*_0_ and *E_g_* are Young’s modulus of shotcrete and reinforcement respectively; *S_g_* and *S_c_* respectively are the cross-sectional areas of reinforcement ribs and shotcrete within the 1 m length selected in the specific calculation.

**Figure 3:**
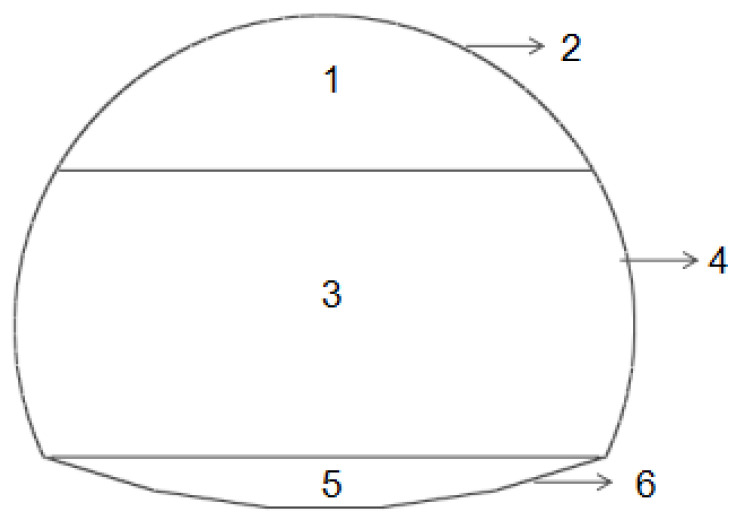
Schematic diagram of construction steps.

In the numerical analysis, the field construction procedures are also simulated as shown in Figure 3. The specific construction steps are carried out as follows: Step 1 (excavating upper bench) → Step 2 (building single-layer lining of upper bench) → Step 3 (excavating lower bench) → Step 4 (building single-layer lining of lower bench) → Step 5 (excavating inverted arch) → Step 6 (building inverted arch).

### 3.2. Numerically Calculated Result

#### 3.2.1. Behavior of Surrounding Rocks

**Figure 4:**
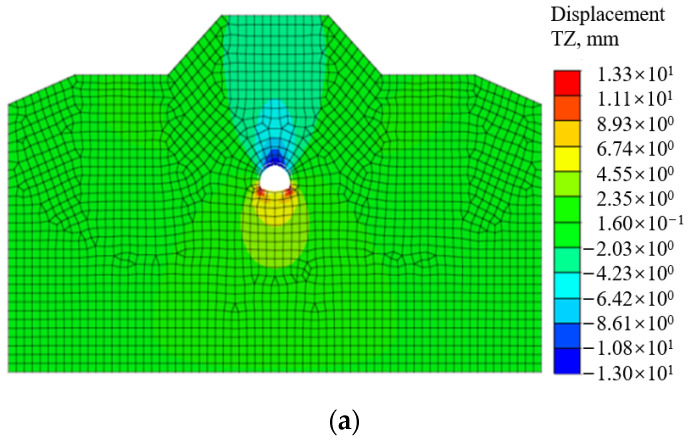

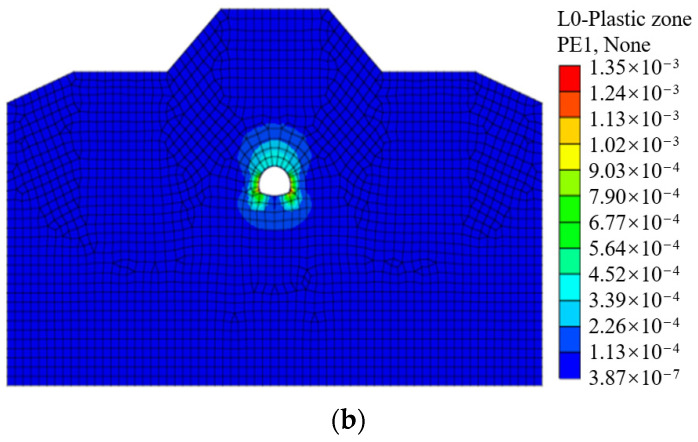
Behavior of surrounding rocks: (**a**) Displacement distribution; (**b**) Plastic zone distribution.

Figure 4 shows the behavior of surrounding rocks after tunnel construction, including the distributions of the displacement and plastic zones. It can be clearly seen that the cumulative settlement of the surrounding rocks at tunnel vault is 13.3 mm, which is significantly smaller than the controlled threshold value of tunnel settlement from 100 mm to 200 mm with the tunnel buried depth from 50 m to 100 m. Moreover, the distributed area of plastic zone in surrounding rocks is relatively small, which indicates that the surrounding rocks is stable after building the single-layer tunnel lining. This behavior implies that the large-span tunnel tends to be stable with employing the single-layer tunnel lining using shotcrete and reinforcement ribs.

#### 3.2.2. Internal Forces of Single-Layer Lining with the Practical Lining Thickness

**Figure 5:**
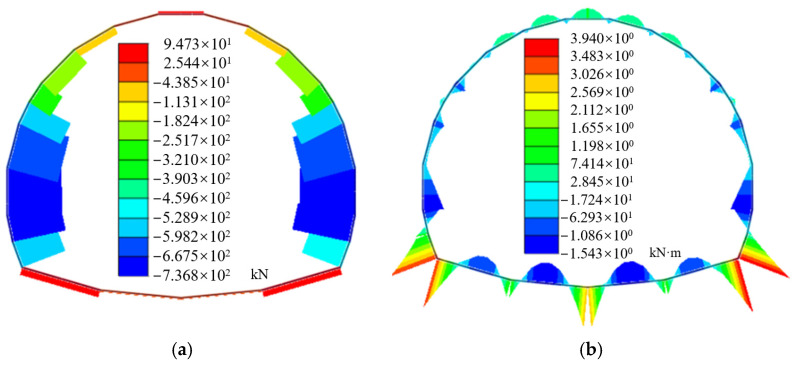
Internal forces of single-layer lining: (**a**) Axial force; (**b**) Bending moment.

Figure 5 demonstrates the axial forces and bending moments of the single-layer tunnel lining after tunnel construction. The axial forces of the single-layer tunnel lining demonstrate that the single-layer tunnel lining is mainly subject to compression, which is decreased from the side wall to the arch spandrel until the arch vault of the large-span tunnel, in which the maximum one is located at the side wall with the value of −737 kN. The invert arch is subject to tension with the maximum axial forces of 94.7 kN. Moreover, the bending moments of the single-layer lining gradually decrease from the side wall to the spandrel of the large-span tunnel, and the maximum positive and negative bending moments appear at arch foot and side wall of single-layer lining respectively.

**Figure 6:**
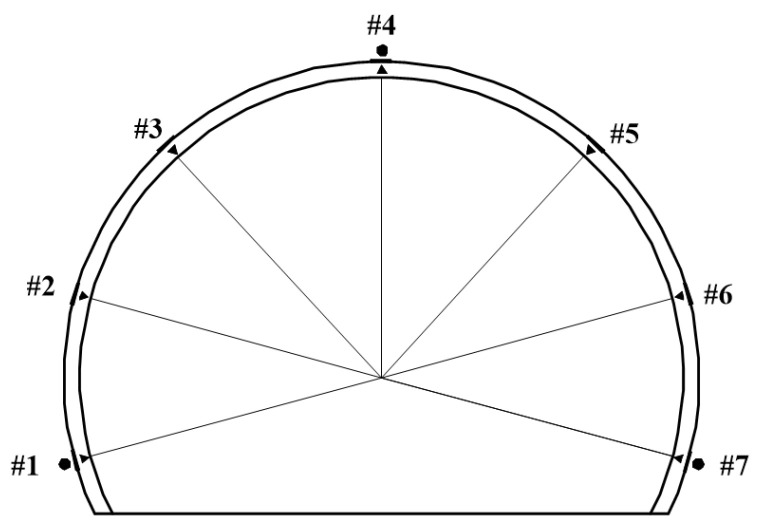
Typical monitoring points in single-layer tunnel lining.

Table 3 demonstrates the numerically obtained stresses of the single-layer tunnel lining at the selected seven typical monitoring points as shown in Figure 6. Moreover, the stresses of the single-layer tunnel lining are calculated using the following Equation (2), in which the axial forces and bending moments of the single-layer tunnel lining are obtained from the numerical simulation, and the designed thickness of the single-layer tunnel lining with 0.15 m is also adopted herein.

(2) $$\sigma =\frac{N}{A}\pm \frac{6M}{bh^{2}}$$

where *σ* is the stress at the outer and inner surfaces of the single-layer tunnel lining, *N* and *M* are the sectional axial force and bending moment of the single-layer tunnel lining respectively, *A* is the sectional area *b* × *h* per unit length in the single-layer tunnel lining, *b* and *h* are the unit length and thickness of the single-layer tunnel lining respectively.

As listed in Table 3, most of the calculated stresses are subject to compressive stresses except that of the arch vault (monitoring point #4) in the single-layer tunnel lining. Moreover, all the numerically calculated stresses are significantly less than those of compressive and tensile strengths of the shotcrete adopted in the single-layer tunnel lining, which implies that there is no damage occurred in the single-layer tunnel lining. Especially, the numerically obtained stresses of the single-layer tunnel lining are similar with the on-site test results at the same position of the single-layer tunnel lining, which verifies the effectiveness of the adopted numerical model.

## 4. Performance of Single-Layer Tunnel Lining Influenced by Lining Thickness and Shallow Buried Depth of the Tunnel

The performance of the single-layer tunnel lining using shotcrete and reinforcement ribs is studied using numerical investigation, taking account of the influence from lining thickness and shallow buried depth of the tunnel, in which the model boundary condition and mesh size are the same with the aforementioned numerical model. Moreover, the behaviors of the single-layer lining and the composite lining structure for supporting the large-span tunnel are compared, which is also expected to reflect the effectiveness of the single-layer tunnel lining using shotcrete and reinforcement ribs in supporting large-span tunnel.

### 4.1. Internal Forces of the Single-Layer Tunnel Lining Influenced by Varied Lining Thicknesses

**Figure 7:**
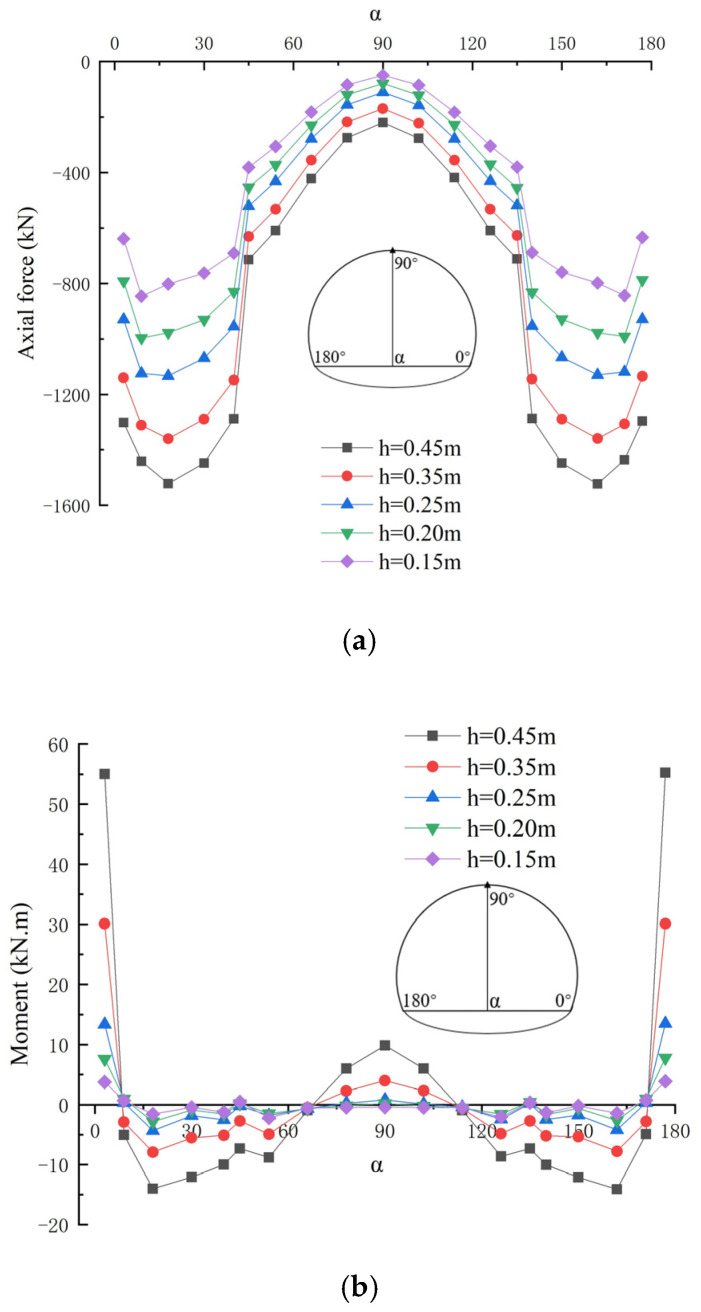
Internal forces of single-layer lining with varied lining thicknesses: (**a**) Axial force; (**b**) Bending moment.

Figure 7 shows the axial forces and bending moments of the single-layer tunnel lining with varied lining thicknesses, in which the factor related to the lining thickness is varied and other parameters remain unchanged, and the varied lining thicknesses are 0.15 m, 0.20 m, 0.25 m, 0.35 m and 0.45 m respectively. It can be clearly seen that the thickness of the single-layer tunnel lining has little influence on the distribution pattern of the axial forces and bending moment in the single-layer tunnel lining, in which the axial forces are gradually decreased from side wall to arch spandrel until arch vault of the single-layer tunnel lining. Moreover, the axial forces of the single-layer tunnel lining are gradually increased with the increasing lining thickness, in which the axial forces at side wall of the single-layer lining are obviously varied with the increasing lining thickness, whereas those of other parts in the single-layer lining are varied little with the increasing lining thickness. The bending moments of the single-layer tunnel lining are also increased with the increasing lining thickness, in which the bending moments at the arch foot and arch vault of the single-layer tunnel lining have the greatest changes. Especially, the maximum positive and negative bending moments appear at the arch foot and side wall of the single-layer tunnel lining respectively.

### 4.2. Internal Forces of Single-Layer Tunnel Lining Influenced by Varied Shallow Buried Depths of the Tunnel

**Figure 8:**
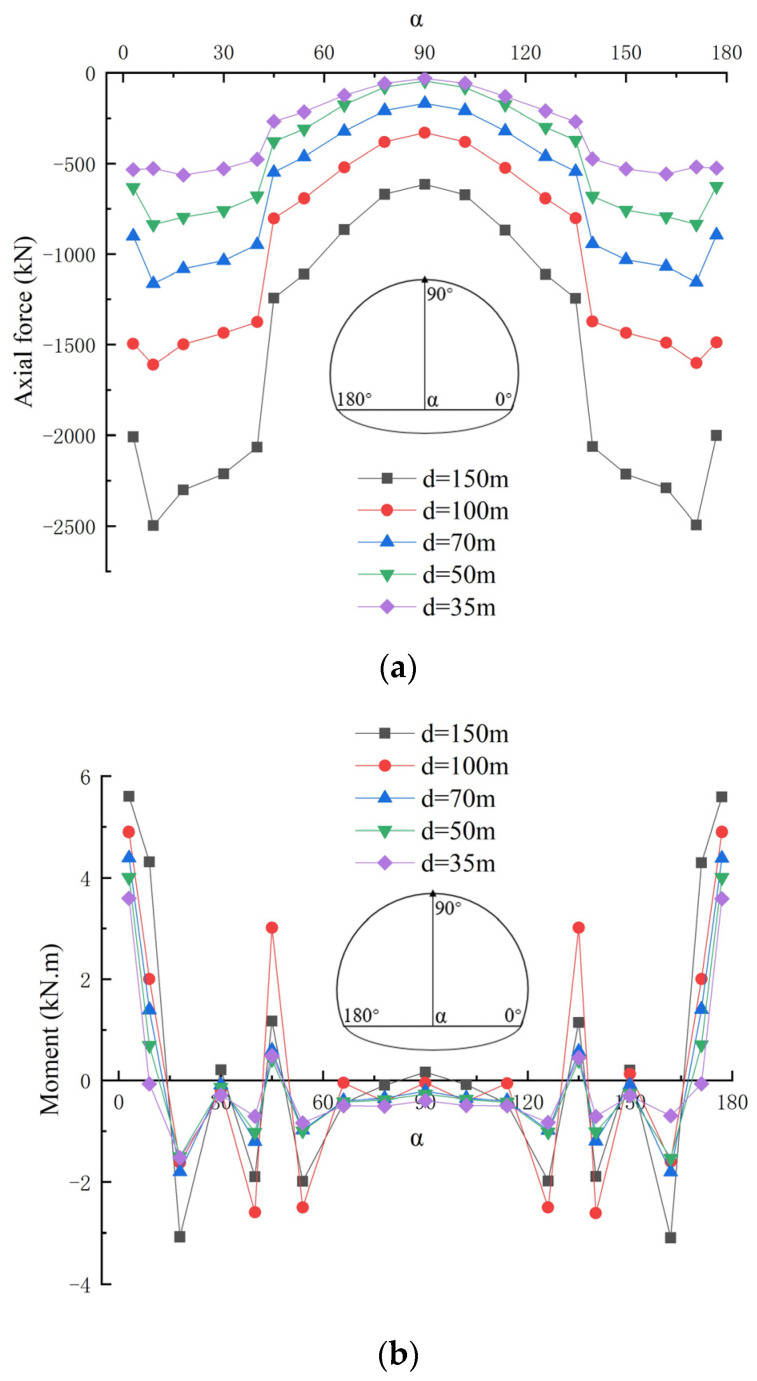
Internal forces of single-layer lining with varied shallow buried depths of the tunnel: (**a**) Axial force; (**b**) Bending moment.

Figure 8 shows the axial forces and bending moments of the single-layer tunnel lining with varied shallow buried depths of the tunnel, in which the factor related to the shallow buried depth of the tunnel is varied and other parameters remain unchanged, and the varied shallow buried depths of the tunnel are 35 m, 50 m, 70 m, 100 m and 150 m respectively. It can be obviously seen that the shallow buried depths of the tunnel have little influence on the axial forces and bending moment distribution pattern of the single-layer tunnel lining, whereas the values of axial forces and bending moments are gradually decreased from side wall to arch spandrel until arch vault of the single-layer tunnel lining. The axial forces of single-layer lining also gradually increase with the increasing shallow buried depth of the tunnel, in which the axial forces of side wall in the single-layer lining are significantly varied with the increase of the tunnel burial depth, whereas those of other parts in the single-layer lining are slowly increased with the increasing shallow buried depth of the tunnel.

Moreover, the bending moment values of the side wall and arch spandrel in the single-layer lining are significantly varied with the increasing shallow buried depth of the tunnel, whereas those of the arch vault in the single-layer lining are small with little variation. Especially, the maximum positive bending moments and minimum negative bending moments appear at the arch foot and arch vault of single-layer lining respectively.

The above investigation demonstrates that the factors related to both the lining thickness and shallow buried depth of the tunnel have great influence on the single-layer tunnel lining with shotcrete and reinforcement ribs, which can be confirmed by the aforementioned safety evaluation of the single-layer tunnel lining using shotcrete and reinforcement ribs. Both the insufficient lining thickness and excessive shallow buried depth of the tunnel can induce damages occurred in the single-layer tunnel lining, due to the stresses of the single-layer tunnel lining exceeding the strength of the shotcrete adopted in the single-layer tunnel lining, and the high-performance concrete thus can be applied as shotcrete in building the single-layer tunnel lining.

### 4.3. Comparative Study on the Performance of Single-Layer Tunnel Lining and Composite Lining Structure

**Figure 9:**
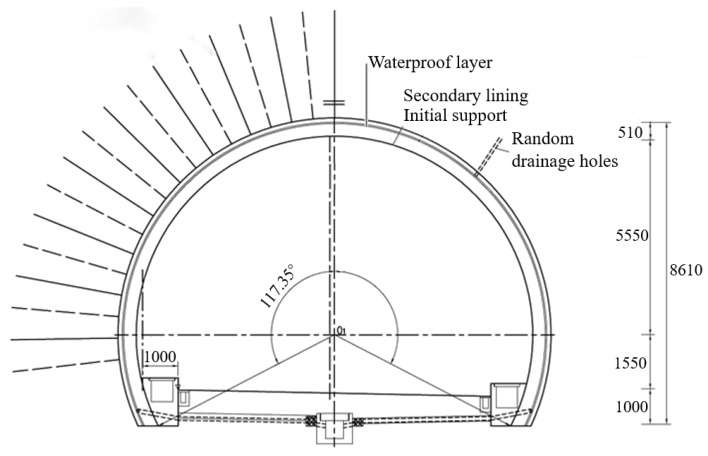
Commonly used composite lining structure (unit: mm).

The comparative study on the performance of the composite lining structure and single-layer tunnel lining using shotcrete and reinforcement ribs and is implemented, in which the composite lining structure of primary lining-waterproof layer-secondary lining are conventionally adopted in tunnel construction. Figure 9 shows the commonly used composite lining structure, in which the primary and secondary linings respectively have the 0.12 m and 0.35 m thicknesses. The single-layer tunnel lining using shotcrete and reinforcement ribs is the same with that adopted in the aforementioned numerical model.

**Figure 10:**
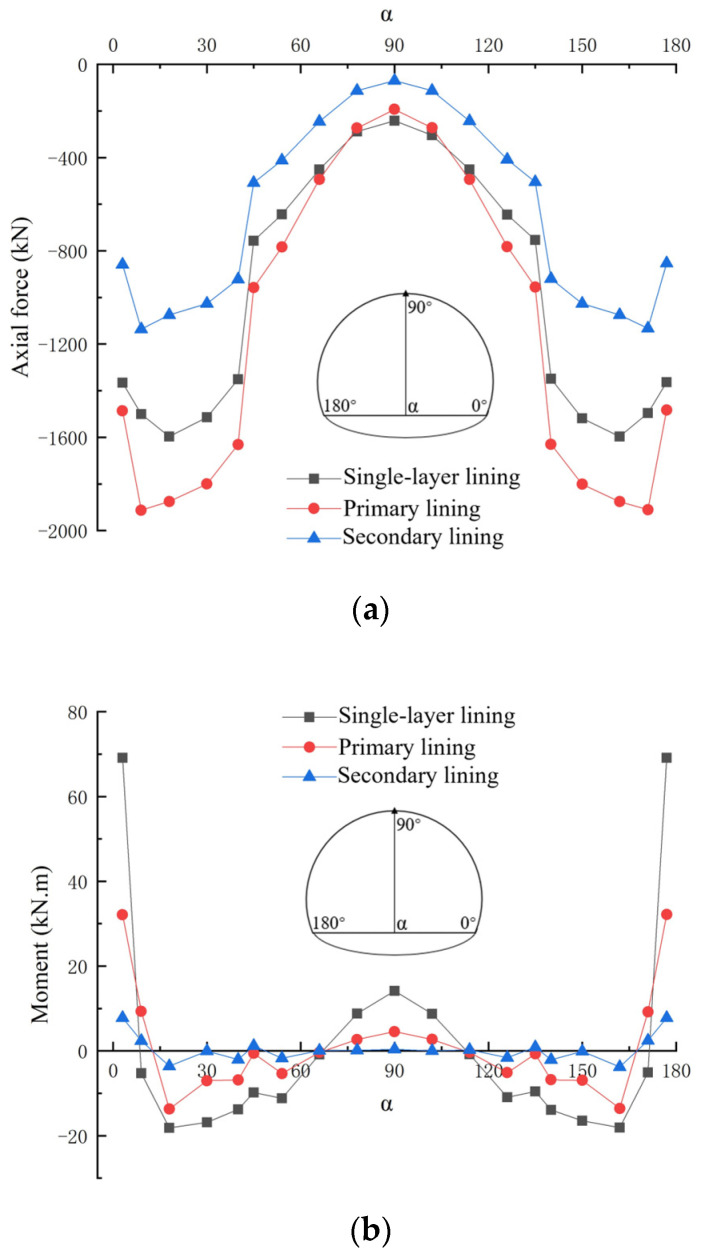
Internal forces of single-layer lining and composite linings: (**a**) Axial force; (**b**) Bending moment.

Figure 10 shows the comparison of the internal forces between the single-layer tunnel lining and composite lining structure. It can be clearly seen that both the axial forces and bending moments of the secondary lining in the composite lining structure are significantly smaller than those of the primary lining in the composite lining structure and the single-layer tunnel lining, in which the axial forces and bending moments of both single-layer tunnel lining and composite lining structure have similar distribution pattern. This implies the reasonability of the single-layer tunnel lining applied in tunnel construction. The axial forces at side wall are greatly changed and those at arch vault have little variation in both single-layer tunnel lining and composite lining structure, in which the axial forces at arch vault in the single-layer tunnel lining are similar with those of the primary lining in composite linings, whereas those at other parts in the single-layer lining are less than those of the primary lining in the composite lining structure. Moreover, the bending moments at arch spandrel are varied little in both single-layer tunnel lining and composite lining structure, and those at arch foot are greatly varied in both single-layer tunnel lining and composite lining structure, in which the maximum positive and negative bending moments respectively appear at arch foot and side wall in both single-layer tunnel lining and composite lining structure.

Based on the above discussion, it is verified that the load carrying mechanism of the single-layer tunnel lining is quite different from that of the composite lining structure, in which the stresses of the composite lining structure are greater than those of the single-layer lining at the same position. This is probably due to the single-layer tunnel lining using shotcrete and reinforcement ribs has the obvious advantage of toughness with significantly absorbing the large deformation of surrounding rocks and improving the ability to resist lining cracking, in which the single-layer tunnel lining shows better integrity and the composite lining structure of the primary lining-waterproof layer-secondary lining has an obvious separation between the primary lining and secondary lining.

## 5. Conclusions

The study of single-layer tunnel lining with shotcrete and reinforcement ribs for supporting the large-span tunnel is implemented in this paper, in which the reinforcement ribs is adopted for improving the stiffness and strength of the single-layer tunnel lining. The study is implemented by numerical analysis, focusing on the safety and performance variation of the single-layer tunnel lining influenced from the varied lining thicknesses and shallow buried depths of the tunnel, and the following conclusions are obtained.

(1) All the calculated stresses of the single-layer tunnel lining using shotcrete and reinforcement ribs are significantly less than the compressive and tensile strengths of shotcrete adopted in the single-layer tunnel lining, which implies that there is no damage occurred in the single-layer tunnel lining. This confirms that the single-layer tunnel lining using shotcrete and reinforcement ribs can be employed for supporting the large-span tunnel, since the single-layer tunnel lining using shotcrete and reinforcement ribs has the obvious advantage of toughness in significantly absorbing the large deformation of surrounding rocks and improving the ability to resist lining cracking.
(2) The numerically obtained stresses of the single-layer tunnel lining using shotcrete and reinforcement ribs are similar with the on-site tested stresses at the same positions in the single-layer tunnel lining, which verifies the effectiveness of the adopted model for numerical analysis.
(3) The thickness of the single-layer tunnel lining and shallow buried depth of the tunnel have great influence on the performance variation of the single-layer tunnel lining using shotcrete and reinforcement ribs. Both the insufficient lining thickness and excessive shallow buried depth of the tunnel can induce damages occurred in the single-layer tunnel lining, due to the stresses of the single-layer tunnel lining exceeding the strength of the shotcrete adopted in the single-layer tunnel lining, and the high-performance concrete is thus recommended as shotcrete in building the single-layer tunnel lining.

## Figures and Tables

**Table 1:** Material parameters of surrounding rocks.

Stratum	Unit Weight (KN/m^3^)	Elastic Modulus (MPa)	Poisson’s Ratio	Friction Angle (°)	Cohesive Force (KPa)
Fully-weathered layer	1700	200	0.33	25	20
Strongly-weathered layer	1800	600	0.22	30	50
Limestone	2500	6000	0.30	39	700

**Table 2:** Material parameters of single-layer lining.

Material	Elastic Modulus (MPa)	Poisson’s Ratio	Unit Weight (KN/m^3^)	Tensile Strength (MPa)	Compressive Strength (MPa)	Diameter of Section (m)
Shotcrete	2.3 × 10^4^	0.2	24	16.7	1.78	/
Reinforcement rib	2 × 10^5^	/	/	300	300	0.22

**Table 3:** Numerical stresses of the monitoring points in the single-layer lining.

Monitoring point	#1	#2	#3	#4	#5	#6	#7
Stress of outer surface (MPa)	−4.93	−3.81	−0.81	0.13	−0.82	−4.15	−4.81
Stress of inner surface (MPa)	−4.15	−3.97	−0.57	0.32	−0.58	−3.69	−5.02

## Data Availability

The data used to support the findings of this study are available from the corresponding author upon request.

## References

[1] Liu S., Fu J., Yang J., Feng H. (2020). Numerical simulation of temperature effects on mechanical behavior of the railway tunnel in tibet. Ksce J. Civ. Eng..

[2] Chen Q.J., Wang J.C., Huang W.M., Yang Z.X., Xu R.Q. (2020). Analytical solution for a jointed shield tunnel lining reinforced by secondary linings. Int. J. Mech. Sci..

[3] Ye Z., Zhang C., Ye Y., Zhu W. (2020). Application of transient electromagnetic radar in quality evaluation of tunnel composite lining. Constr. Build. Mater..

[4] Rosso M.M., Marasco G., Aiello S., Aloisio A., Chiaia B., Marano G.C. (2023). Convolutional networks and transformers for intelligent road tunnel investigations. Comput. Struct..

[5] Ding H., Tong L.H., Xu C., Hu W. (2020). Aseismic performance analysis of composite lining embedded in saturated poroelastic half space. Int. J. Geomech..

[6] Zhu Y., Liu C. (2020). Study on parameter influence of new composite lining of water conveyance tunnel under high internal water pressure. Front. Earth Sci..

[7] Zhu Y., Liu C., Yin X., Zhang J. (2022). Analysis of the internal force and deformation characteristics of double-layer lining structure of water conveyance tunnel. Geofluids.

[8] de Alencar Monteiro V.M., de Andrade Silva F. (2021). On the design of the fiber reinforced shotcrete applied as primary rock support in the Cuiabá underground mining excavations: A case study. Case Stud. Constr. Mater..

[9] He B.-G., Zhang Y., Zhang Z.-Q., Feng X.-T., Sun Z.-J. (2021). Model test on the behavior of tunnel linings under earth pressure conditions and external water pressure. Transp. Geotech..

[10] Leung C.K.Y., Lai R., Lee A.Y.F. (2005). Properties of wet-mixed fiber reinforced shotcrete and fiber reinforced concrete with similar composition. Cem. Concr. Res..

[11] Franzén T. (1993). Shotcrete for rock support: A summary report on the state of the art in 15 countries. Tunn. Undergr. Space Technol..

[12] Li P., Wang F., Fang Q. (2018). Undrained analysis of ground reaction curves for deep tunnels in saturated ground considering the effect of ground reinforcement. Tunn. Undergr. Space Technol..

[13] Galan I., Baldermann A., Kusterle W., Dietzel M., Mittermayr F. (2019). Durability of shotcrete for underground support—Review and update. Constr. Build. Mater..

[14] Cui S., Xu D.-L., Liu P., Ye Y. (2016). Exploratory study on improving bond strength of shotcrete in hot and dry environments of high geothermal tunnels. Ksce J. Civ. Eng..

[15] Liu X., Sun Q., Song W., Bao Y. (2022). Numerical modeling and parametric study of hybrid fiber-rebar reinforced concrete tunnel linings. Eng. Struct..

[16] Liu X., Sun Q., Song W., Bao Y. (2023). Structural behavior of reinforced concrete tunnel linings with synthetic fibers addition. Tunn. Undergr. Space Technol..

[17] Massone L.M., Nazar F. (2018). Analytical and experimental evaluation of the use of fibers as partial reinforcement in shotcrete for tunnels in Chile. Tunn. Undergr. Space Technol..

[18] Avanaki M.J., Abedi M., Hoseini A. (2020). Experimental and numerical-based design of hybrid steel fibre-reinforced concrete tunnels. Mag. Concr. Res..

[19] Lü W., Sun H. (2020). Study on support characteristic curve of primary support structures in underground excavation considering bond-slip behavior. Adv. Struct. Eng..

[20] Qi Z., Chen W., Zhang L., Huang Z. (2020). An integrated design method for functional cementitious composites (FCC). Constr. Build. Mater..

[21] Nie H., Gu S. (2020). Ultimate bearing capacity analysis of CFRP-strengthened shield segments using bonding slip behavior experiments. Materials.

[22] Li X., Zhang T., Ding Z., Yang X., Wen J. (2020). Numerical analysis of normal concrete lining strengthening methods under different damage levels. Struct. Infrastruct. Eng..

[23] Kong D., Xu Y., Song C. (2020). Dynamic response of composite steel lining structure under blast loading. Shock. Vib..

[24] Li H.Y., Wu Y.F., Zhou A.X., Lu F., Lei Z.C., Zeng B.W., Zhu K.C. (2023). Cracking pattern and bearing capacity of steel fiber-reinforced concrete single-layer tunnel lining. Sustainability.

[25] Zhao Y., Liu C., Zhang Y., Yang J., Feng T. (2019). Damaging behavior investigation of an operational tunnel structure induced by cavities around surrounding rocks. Eng. Fail. Anal..

[26] Zhang Y.X., Yang J.S., Yang F. (2015). Field investigation and numerical analysis of landslide induced by tunneling. Eng. Fail. Anal..

[27] Zhang Y., Shi Y., Zhao Y., Fu L., Yang J. (2017). Determining the cause of damages in a multiarch tunnel structure through field investigation and numerical analysis. J. Perform. Constr. Facil..

